# Multipotent adult progenitor cells grown under xenobiotic-free conditions support vascularization during wound healing

**DOI:** 10.1186/s13287-020-01912-3

**Published:** 2020-09-07

**Authors:** Bart Vaes, Ellen Van Houtven, Ellen Caluwé, Aernout Luttun

**Affiliations:** 1 ReGenesys BVBA, Heverlee, Belgium; 2 Center for Molecular and Vascular Biology, Endothelial Cell Biology Unit, KU Leuven, Campus Gasthuisberg, Onderwijs & Navorsing 1, Herestraat 49, B-3000 Leuven, Belgium

**Keywords:** Stem cells, Xenobiotic-free culture media, Wound healing

## Abstract

**Background:**

Cell therapy has been evaluated pre-clinically and clinically as a means to improve wound vascularization and healing. While translation of this approach to clinical practice ideally requires the availability of clinical grade xenobiotic-free cell preparations, studies proving the pre-clinical efficacy of the latter are mostly lacking. Here, the potential of xenobiotic-free human multipotent adult progenitor cell (XF-hMAPC®) preparations to promote vascularization was evaluated.

**Methods:**

The potential of XF-hMAPC cells to support blood vessel formation was first scored in an in vivo Matrigel assay in mice. Next, a dose-response study was performed with XF-hMAPC cells in which they were tested for their ability to support vascularization and (epi) dermal healing in a physiologically relevant splinted wound mouse model.

**Results:**

XF-hMAPC cells supported blood vessel formation in Matrigel by promoting the formation of mature (smooth muscle cell-coated) vessels. Furthermore, XF-hMAPC cells dose-dependently improved wound vascularization associated with increasing wound closure and re-epithelialization, granulation tissue formation, and dermal collagen organization.

**Conclusions:**

Here, we demonstrated that the administration of clinical-grade XF-hMAPC cells in mice represents an effective approach for improving wound vascularization and healing that is readily applicable for translation in humans.

## Background

Wound healing is one of the areas of unmet clinical need for which stem cell therapy may offer a solution. Although wound healing is a naturally occurring process able to restore injured tissues, there is an increasing number of patients suffering from chronic wounds that are refractory to healing. There may be various causes for disturbed wound healing such as diabetes, vascular disease, or old age [1], and given the longer life expectancy and the sharply rising incidence of age-related conditions, the prevalence of chronic wounds is expected to increase dramatically in the following years.

Cutaneous wound healing occurs in four partially overlapping phases [2, 3]. During the hemostatic phase, the clotting cascade is activated to prevent excessive blood loss. During the inflammatory phase, neutrophils, lymphocytes, macrophages, and mast cells move to the wound site to clear up damaged material and bacteria. During the proliferation or granulation tissue formation phase, a provisional collagen matrix is deposited by (myo) fibroblasts, ingrowth of new blood vessel ensues, re-epithelialization by keratinocytes is stimulated, and wound contraction by myofibroblasts occurs. Finally, during the resolution or maturation phase, collagen is being remodeled and the wound is fully closed.

New blood vessel growth in wounds is one of the central events that orchestrate the healing process. Hence, many therapeutic approaches have been designed to improve wound vascularization. Cell therapy is considered as a promising option for wound repair as multiple reports have demonstrated that stem cells can promote wound healing by triggering vascularization as one of the mechanisms underlying their beneficial effect [4]. While many studies have been carried out using mesenchymal stromal cells (MSCs), other cell types may have therapeutic potential as well [4]. Human multipotent adult progenitor cells (hMAPCs) are bone marrow-derived adherent stem cells with high proliferative and regenerative capacity in pre-clinical disease models such as graft versus host disease [5], spinal cord injury [6], traumatic brain injury [7], organ transplantation [8], hind limb ischemia [9], and lymphedema [10]. The most important mechanisms underlying this regenerative capacity are known to be the immunomodulatory and pro-angiogenic features of the cells. MAPC cells have been reported to induce revascularization in in vivo pre-clinical models of hind limb ischemia [9], orthopedic defects [11], and islet transplantation in diabetic mice [12]. Interestingly, the study by LoGuidice et al. reported a higher angiogenic potential for MAPC cells than for MSCs. This result is in line with the observation that MAPC cells in Matrigel transplanted under the skin of nude mice led to more and better blood vessels compared to MSCs [13]. While MAPC cells have the intrinsic potential to directly contribute to new blood vessels by vascular differentiation and incorporation, they mostly have a pro-vascularization effect by trophic stimulation. That such mechanisms are indeed relevant for wound healing has recently been confirmed in a study showing that MAPC cells improved wound healing by enhancing angiogenesis as well as lymphangiogenesis [10].

Although for chronic ulcers, autologous cell therapy may be used, the time needed to expand the cells and perform quality analysis does not allow such a strategy for other types of wounds such as burns or trauma. The high expansion potential and low immunogenicity of MAPC cells allow for the development of an off-the-shelf allogeneic product. One of the drawbacks in current expansion procedures for cell therapy is the use of animal-derived serum in the medium because of limited availability, batch variability, and ethical concerns [14]. It is thus desirable to develop stem cell expansion procedures in which the serum-containing media are being replaced by serum-free media or xenobiotic-free (XF) media in case no animal-derived compounds are present.

It has previously been reported that hMAPC cells expanded under XF conditions (XF-hMAPC cells) maintained typical MAPC characteristics including proliferation potential, marker expression, and in vitro immunosuppressive and pro-angiogenic capacity [15]. In the current study, we evaluated whether the XF-hMAPC cells have regenerative capacity in vivo in a mouse model of full-thickness splinted wounds. We demonstrate that XF-hMAPC cells had a dose-dependent effect on multiple wound healing parameters, including wound closure, vascularization, re-epithelialization, and collagen organization.

## Methods

### Cell derivation and culture

hMAPC cells were isolated by ReGenesys BVBA (Athersys affiliate; Heverlee, Belgium) from a single bone marrow aspirate from a healthy 35-year-old female donor (Lonza, Belgium; after obtaining informed consent and ethical approval). Cells were cultured on Corning**®** CellBind**®** surface culture flasks in off-the-shelf XF media supplemented with additional MAPC growth components, maintained under low oxygen tension in a humidified atmosphere of 5% CO_2_, cultured to subconfluence in XF-hMAPC culture media, and passaged by using TrypLE Select (Thermo Fisher Scientific Life Sciences, Waltham, MA). Cells were phenotyped in terms of marker expression and differentiation potential, as described [15].

### In vivo Matrigel implantation assay

As MAPC cells express low levels of Major Histocompatibility Complex-I and—consequently—could be susceptible to natural killer cell-mediated clearance, all mice (including phosphate-buffered saline or PBS controls) were injected intraperitoneally with anti-asialo GM1 antibodies (Wako Chemicals, Osaka, Japan) 1–2 h before transplantation and every 10 days thereafter. These antibodies selectively eliminate natural killer cells without affecting macrophage or lymphocyte function [16]. Eight-week-old female athymic nude mice (Envigo, Horst, The Netherlands; a total of 11 mice was used for this part of the study) were anesthetized with isoflurane and each injected subcutaneously in the back with a mixture of 50 μL cell suspension (4 × 10^6^ cells in PBS), 10 μL of concentrated solution of angiogenic growth factors (final concentrations corresponding to 300 ng/mL rh-VEGF_165_ and 700 ng/mL rh-bFGF), and 240 μL of cold growth factor-reduced Matrigel. As a control, 50 μL of PBS was mixed with concentrated growth factor solution and cold Matrigel and injected subcutaneously. After 21 days, Matrigel plugs were dissected out, photographed using a digital camera (Axiocam ERc 5S, Zeiss), fixed in zinc-paraformaldehyde, and processed for paraffin embedding and sectioning.

### In vivo wound healing assay

Mice were injected intraperitoneally with anti-asialo GM1 antibodies 1–2 h before wounding. At day 0, 8-week-old athymic nude Foxn1 females (Envigo) were anesthetized with an intraperitoneal injection of ketamine (100 mg/kg) and xylazine (10 mg/kg). Under sterile and temperature-controlled (37 °C) conditions, standardized full-thickness wounds were made with a 0.5 cm biopsy puncher (Stiefel Laboratories, Offenbach am Main, Germany) on the back of the mouse in the mid-dorsal region. A silicone ring was fixed (using Histoacryl tissue adhesive, Braun, Diegem, Belgium) and sutured around the wound, and wounds were treated with Plasma-Lyte control (“CTRL”) or 2.5 × 10^5^, 5.0 × 10^5^, or 1.0 × 10^6^ XF-cultured hMAPC cells (XF1, XF2, or XF3, respectively, suspended in Plasma-Lyte), locally pipetted onto the wound in a volume of 30 μL. From a previous study [10], we showed that this application method ensures a homogeneous distribution of cells across the wound bed. An occlusive dressing (Tegaderm™, 3 M, Diegem, Belgium) was used to keep the wound moist. Every other day, the dressing was renewed under isoflurane anesthesia, and pictures were taken using a NikonD1 camera and Camera-Control-Pro software. Wound size was measured by a blinded observer using ImageJ software and was expressed as the % versus the size at day 0 for each individual mouse. At 5 or 10 days after wounding, mice were euthanized and square skin fragments including the circular wound area and a rim of normal skin were dissected out and post-fixed overnight using zinc-paraformaldehyde. Following fixation, skin fragments were separated in two equal pieces at the wound midline and processed for paraffin embedding and sectioning. For the first part of the study related to the effects on early wound healing, a total of 59 mice were used; for the second part of the study related to the late stages of wound healing, 47 mice were used. We chose to exclusively use female mice in this model since male mice tended to frequently remove the occlusive dressing resulting in wound bed drying.

### Histology and morphometry

One or two series of 10 slides were covered with twelve paraffin serial sections with a thickness of 7 μm without trimming in between each collected series of 10 sections. Paraffin sections were dehydrated, stained, and photographed using a Zeiss Axiovert 200 M microscope, a Zeiss Axio Imager Z1 microscope equipped with a Zeiss MRc5 camera or a Leica Leitz DMRBE microscope equipped with a Zeiss MRc5 camera and Axiovision 4.8 software. Images were cropped, pseudo-colored, and contrast-adjusted using Photoshop (Adobe). Using ImageJ software, morphometric analysis for the in vivo Matrigel study was done on 2 areas/section from 3 to 4 different serial sections (each 140 μm apart) for each Matrigel. Host blood vessel ingrowth, vessel size distribution, and maturation (by coverage with smooth muscle cells (SMCs) or fibrillar collagen) were quantified by determining the fractional area positive for mouse (m)CD31, by measuring cross-sectional vessel area, or by counting the percentage of α-smooth muscle-actin (αSMA)^+^mCD31^+^ vessels or the amount of perivascular fibrillar collagen deposited, respectively, on sections co-stained with rat anti-mouse CD31 (BD Pharmingen 557355; specific for mouse cells) and mouse monoclonal αSMA (Cy3-conjugated; Sigma C6198) or stained with Sirius red, as described [17]. The presence of human CD34^+^ cells was traced on sections stained with mouse anti-CD34 antibody (BD Pharmingen 555821; specific for human cells). The fibrillar collagen fractional area was determined on Sirius red-stained sections analyzed by brightfield microscopy. All morphometric analyses were performed in a blinded fashion.

For the wound healing study, morphometric analysis was done on 2–4 areas/section from 3 to 5 different consecutive serial sections (each 70 μm apart) from both wound halves. Wound re-epithelialization (expressed as %) was measured on pancytokeratin (PCK; Sigma C2562)-stained (day 5 post-wounding) or hematoxylin/eosin (H&E)-stained (day 10 post-wounding) sections by measuring the cumulative distance covered by the epithelial tongues from both wound borders divided by the total wound length. Granulation tissue formation (expressed as arbitrary units) was quantified at day 5 post-wounding on H&E-stained sections by determining the area of granulation tissue, normalized for the wound length. Vascular ingrowth (expressed as %) was determined on CD31-stained sections by measuring the cumulative CD31^+^ vessel ingrowth distance from both wound borders divided by the total wound length. Wound vascularity (expressed as number/area) was determined by counting the number of CD31^+^ or αSMA^+^ vessels in the wound borders (day 5) or in the wound center (day 10). Wound vessel maturation (expressed as %) was determined by calculating the ratio of αSMA-coated vessels per CD31^+^ vessels on CD31/αSMA co-stained cross-sections. Fibrillar collagen deposition or organization (revealed by red birefringence) was expressed as % and determined on day 10 after wounding by measuring the area taken up by Sirius red-staining relative to the total wound area upon brightfield or polarized light microscopy, respectively. The fraction of organized (red birefringent) collagen was calculated as a ratio of organized versus total fibrillar collagen and expressed as %. The presence of myofibroblasts (defined as αSMA^+^ cells not directly associated with CD31^+^ vessels) was determined on CD31/αSMA co-stained cross-sections. All morphometric analyses were performed in a blinded fashion.

### Statistical analysis

Quantitative data represent mean ± S.E.M. “*n*” represents the number of independent biological replicates on which statistical tests were performed. Normality of the data was confirmed by the Shapiro-Wilk test. Two-group comparisons were performed by unpaired Student’s *t* test. Multiple-group comparisons were done by 1-way ANOVA with Tukey’s or Dunnett’s post hoc test. Wound size evolution in time was evaluated by repeated measures ANOVA, followed by Tukey’s post hoc test. Data were considered significant if the *P* value was less than 0.05. All analyses were performed with Graphpad Prism (version 7.0).

## Results

### XF-hMAPC cells formed an elaborate and mature tubular network in Matrigel in vivo

**Fig. 1:**
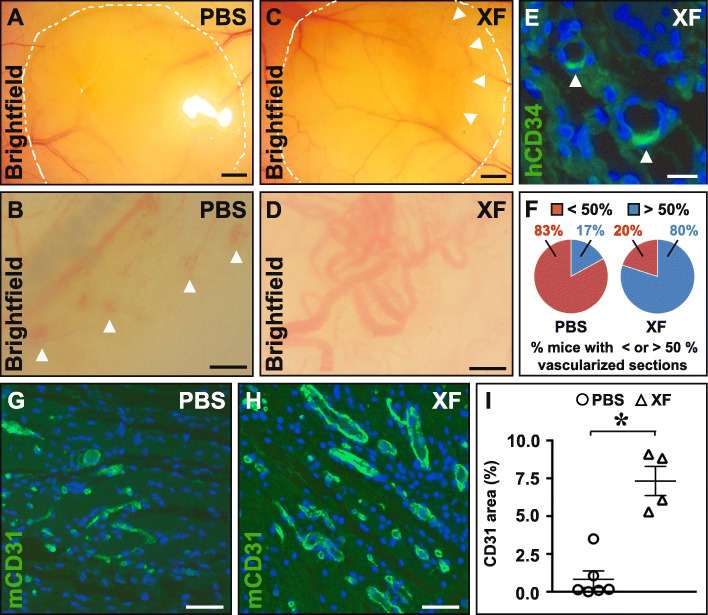
XF-hMAPC cells induced an elaborate host vascular network in an in vivo Matrigel implantation assay. **a**–**d** Brightfield images of implants at lower (**a**, **c** implant borders are lined by dashed white lines) and higher magnification (**b**, **d**) containing PBS (**a**, **b**) or XF-hMAPC cells (“XF”; **c**, **d**). Clearly, vascularized areas and vascular leakage are indicated by white arrowheads in panel **c** or **b**, respectively. **e** Cross-section of an XF implant stained with anti-human (h)CD34 in green. Positive cells are indicated by white arrowheads. **f** Pie diagrams representing the fraction of mice with more (blue) or less (red) than 50% of the examined sections containing vessels for the PBS (left), or XF (right) group. **g**–**i** Cross-sections stained for mouse (m)CD31 in green for the PBS (**g**; open circles in **i**; *n* = 6), or XF (**h**; open triangles in **i**; *n* = 4) groups and the corresponding quantification in **i**. Data represent the mean fractional CD31^+^ area expressed as % ± S.E.M. (*P < 0.05 versus PBS by unpaired Student’s *t* test). DAPI was used as nuclear counterstaining (in blue) in **e**, **g**, **h**. Magnifications at which pictures were taken: × 10 in **g**, **h**; × 40 in **e**. Scale bars: 1.3 mm in **a**, **c**; 200 μm in **b**, **d**; 50 μm in **g**, **h**; and 20 μm in **e**

**Fig. 2:**
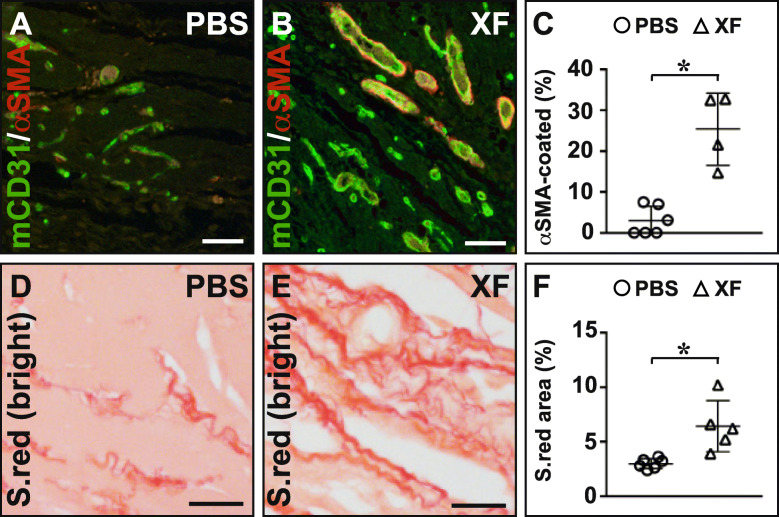
XF-hMAPC cells induced a mature host vascular network in an in vivo Matrigel implantation assay. **a**–**c** Cross-sections stained for mouse (m)CD31 in green and α-smooth muscle-actin (αSMA) in red for the PBS (**a**; open circles in **c**; *n =* 6) or XF-hMAPC cell (“XF”; **b**; open triangles in **c**; *n* = 4) group and the corresponding quantification in **c**. Data represent the mean fraction of αSMA^+^ vessels expressed as % of CD31^+^ vessels ± S.E.M. (*P < 0.05 versus PBS by unpaired Student’s *t* test). **d**–**f** Cross-sections stained for Sirius red (“S.red”) and photographed in brightfield for the PBS (**d**; open circles in **f**; *n* = 6), or XF-hMAPC (**e**; open triangles in **f**; *n* = 5) group and the corresponding quantification in **f**. Data represent mean fractional Sirius red^+^ area expressed as % ± S.E.M. (*P < 0.05 versus PBS by unpaired Student’s *t* test). Panels **a**, **b** correspond to panels **g**, **h** of Fig. 1. Magnifications at which pictures were taken: × 10 in **a**, **b**; × 20 in **d**, **e**. Scale bars: 50 μm in **a**, **b**; 20 μm in **d**, **e**

Upon Matrigel implantation in vivo, compared to implants containing PBS, XF-hMAPC cell-loaded implants were clearly more vascularized as evident from the yellow-orange areas within the implants (Fig. 1a, c). At higher magnification, vessels in PBS-containing but not those in XF-hMAPC-containing implants showed leakage and vessels in XF-hMAPC implants seemed larger (Fig. 1b, d). XF-hMAPC cells gave rise to CD34^+^ endothelial cells (Fig. 1e); however, their direct contribution to vascular structures was very limited, suggesting that the cells mainly had trophic effects on the ingrowing mouse host vasculature. Consistent with the macroscopic observations, implants with XF-hMAPC cells were more vascularized than PBS-implants as shown by a higher fraction of mice with more than half of the Matrigel sections containing vessels (Fig. 1f). XF-hMAPC-seeded Matrigels had larger vascular fractional areas and a higher degree of SMC coverage in their implants than the PBS group (Figs. 1g–i and 2a–c). Furthermore, significantly more perivascular fibrillar collagen was deposited in XF-hMAPC-containing implants compared to PBS implants (Fig. 2d–f). Thus, upon implantation in a Matrigel plug in vivo, XF-hMAPC cells boosted the ingrowth of host vessels, which acquired maturity features.

### XF-hMAPC cells dose-dependently improved early vascularization and healing of wounds

**Fig. 3:**
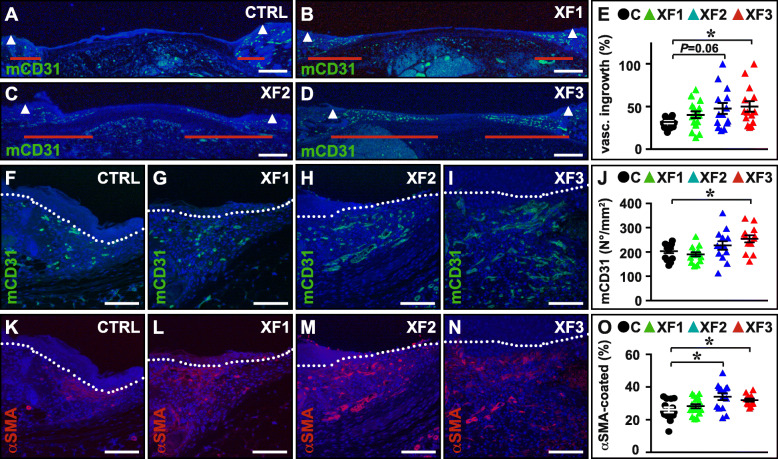
XF-hMAPC cells dose-dependently induced an elaborate and mature vascular network in the wound edges early during wound healing. **a**–**e** Cross-sections representing an overview of the wound bed stained for mouse (m)CD31 in green for the Plasma-Lyte control (“CTRL”; **a**; black circles in **e**; *n* = 15), low-dose XF-hMAPC cell (“XF1”; **b**; green triangles in **e**; *n* = 16), middle-dose XF-hMAPC cell (“XF2”; **c**; blue triangles in **e**; *n* = 14), and high-dose XF-hMAPC cell (“XF3”; **d**; red triangles in **e**; *n* = 14) groups and the corresponding quantification in E. Data represent the vascular ingrowth distance relative to the wound length expressed as % ± S.E.M. (*P < 0.05 versus indicated condition by one-way ANOVA). Red horizontal lines in **a**–**d** indicate distance covered by the vascular fronts, white arrowheads indicate wound borders. **f**–**j** Cross-sections zooming in on the wound edges stained for mCD31 in green for CTRL (**f**; black circles in **j**; *n* = 14), XF1 (**g**; green triangles in **j**; *n* = 14), XF2 (**h**; blue triangles in **j**; *n* = 13), and XF3 (**i**; red triangles in **j**; *n* = 13) groups and the corresponding quantification in **j**. Data represent the mean number of mCD31^+^ vessels expressed per area (in mm^2^) ± S.E.M. (*P < 0.05 versus indicated condition by one-way ANOVA). **k**–**o** Cross-sections (same as in **f**–**i**) zooming in on the wound edges stained for α-smooth muscle-actin (αSMA) in red for CTRL (**k**; black circles in **o**; *n* = 14), XF1 (**l**; green triangles in **o**; *n* = 16), XF2 (**m**; blue triangles in **o**; *n* = 14), and XF3 (**n**; red triangles in **o**; *n* = 12) groups and the corresponding quantification in **o**. Data represent the mean fraction of αSMA^+^ vessels expressed as % of CD31^+^ vessels ± S.E.M. (*P < 0.05 versus indicated condition by one-way ANOVA). Dermo-epidermal junction is indicated by white dotted lines in **f**–**i** and **k**–**n**. DAPI was used as nuclear counterstaining (in blue) in **a**–**d**, **f**–**i**, and **k**–**n**. Magnifications at which pictures were taken: × 2.5 in **a**–**d**; × 20 in **f**–**i**, **k**–**n**. Scale bars: 500 μm in **a**–**d**, 100 μm in **f**–**i** and **k**–**n**

While Matrigel implantation is a frequently used assay to evaluate blood vessel growth and maturation, it represents a rather artificial and less robust model [18]. We next sought to determine and confirm their efficiency to support blood vessel growth in a physiologically more relevant model, i.e., skin wound healing. First, we determined the effect of XF-hMAPC cells in the early stages after wounding in a dose-response set-up, using three doses (2.5 × 10^5^ or “XF1,” 5.0 × 10^5^ or “XF2,” and 1.0 × 10^6^ or “XF3”) and Plasma-Lyte vehicle control (“CTRL”) as reference condition. Initial wound sizes measured immediately after wound infliction were similar across the different treatment conditions (expressed as % versus the inner area of the ring sutured around the wound: 26 ± 2 for CTRL (*n* = 15), 27 ± 2 for XF1 (*n* = 16), 30 ± 2 for XF2 (*n* = 14), and 31 ± 3 for XF3 (*n* = 14; *P* > 0.05 by one-way ANOVA). Local application of XF-hMAPC cells had a limited dose-dependent effect on wound closure by contraction on day 5 after wounding, which was only statistically significant for the highest cell dose (wound size in % versus day 0: 79 ± 3 for CTRL (*n* = 15), 72 ± 3 for XF1 (*n* = 16), 71 ± 4 for XF2 (*n* = 14), and 63 ± 5 for XF3 (*n* = 14; *P* < 0.05 versus CTRL by one-way ANOVA). XF-hMAPC cells significantly accelerated the ingrowth of CD31^+^ blood vessels towards the wound center (Fig. 3a–e) and increased the number of CD31^+^ blood vessels in the wound edges at 5 days post-wounding (Fig. 3f–j), both in a dose-dependent manner. Furthermore, in accordance with the Matrigel implantation assay results, a significant fraction of these ingrowing vessels was stabilized by αSMA coating, which was more evident in the higher cell doses (Fig. 3k–o). Thus, XF-hMAPC transplantation significantly promoted the ingrowth and maturation of blood vessels in the wound bed.

**Fig. 4:**
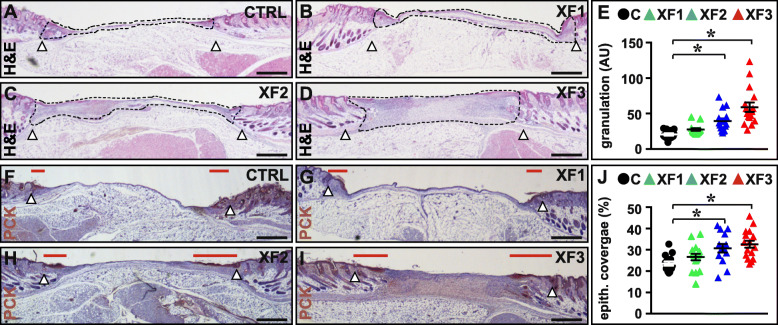
XF-hMAPC cells dose-dependently induced granulation and re-epithelialization early during wound healing. **a**–**e** Cross-sections representing an overview of the wound bed stained with hematoxylin/eosin (H&E) for the Plasma-Lyte control (“CTRL”; *A*; black circles in **e**; *n* = 14), low-dose XF-hMAPC cell (“XF1”; **b**; green triangles in **e**; *n* = 13), middle-dose XF-hMAPC cell (“XF2”; **c**; blue triangles in **e**; *n* = 15), and high-dose XF-hMAPC cell (“XF3”; **d**; red triangles in **e**; *n* = 16) groups and the corresponding quantification in **e**. Data represent mean granulation tissue amount, expressed as arbitrary units ± S.E.M. (*P < 0.05 versus indicated condition by one-way ANOVA). Granulation tissue area is lined by black dashed lines in **a**–**d**. **f**–**j** Cross-sections representing an overview of the wound bed stained for pancytokeratin (PCK) for the CTRL (**f**; black circles in **j**; *n* = 15), XF1 (**g**; green triangles in **j**; *n* = 16), XF2 (**h**; blue triangles in **j**; *n* = 14), and XF3 (**i**; red triangles in **j**; *n* = 16) groups and the corresponding quantification in **j**. Data represent mean re-epithelialization distance from the wound edges relative to the wound length expressed as % ± S.E.M. (*P < 0.05 versus indicated condition by one-way ANOVA). Red horizontal lines in **f**–**i** indicate distance covered by the neo-epidermis. Outer wound edges are indicated by arrowheads in **a**–**d** and **f**–**i**. Hematoxylin was used as nuclear counterstaining (in blue) in **a**–**d** and **f**–**i**. Magnifications at which pictures were taken: × 2.5 in **a**–**d**, **f**–**i**. Scale bars: 500 μm in **a**–**d** and **f**–**i**

We and others previously showed that increased wound vascularization is associated with improved healing parameters, including granulation tissue formation [19] and epidermal regeneration [17]. Accordingly, compared to vehicle-treatment, XF-hMAPC cells significantly and dose-dependently increased granulation tissue formation in the gap created by the full-thickness wound (Fig. 4a–e). As in this splinted model wounds not only close by contraction but also by regenerative healing (i.e., re-epithelialization), we next evaluated the effect of XF-hMAPC transplantation on the advancement of the PCK^+^ epithelial tongues on both edges of the wound and found a dose-dependent and significant improvement in this parameter (Fig. 4f–j). Thus, the beneficial and dose-dependent improvement in wound vascularization was accompanied by improved healing in the early stages of the healing process.

### XF-hMAPC cells dose-dependently improved late vascularization and healing of wounds

**Fig. 5:**
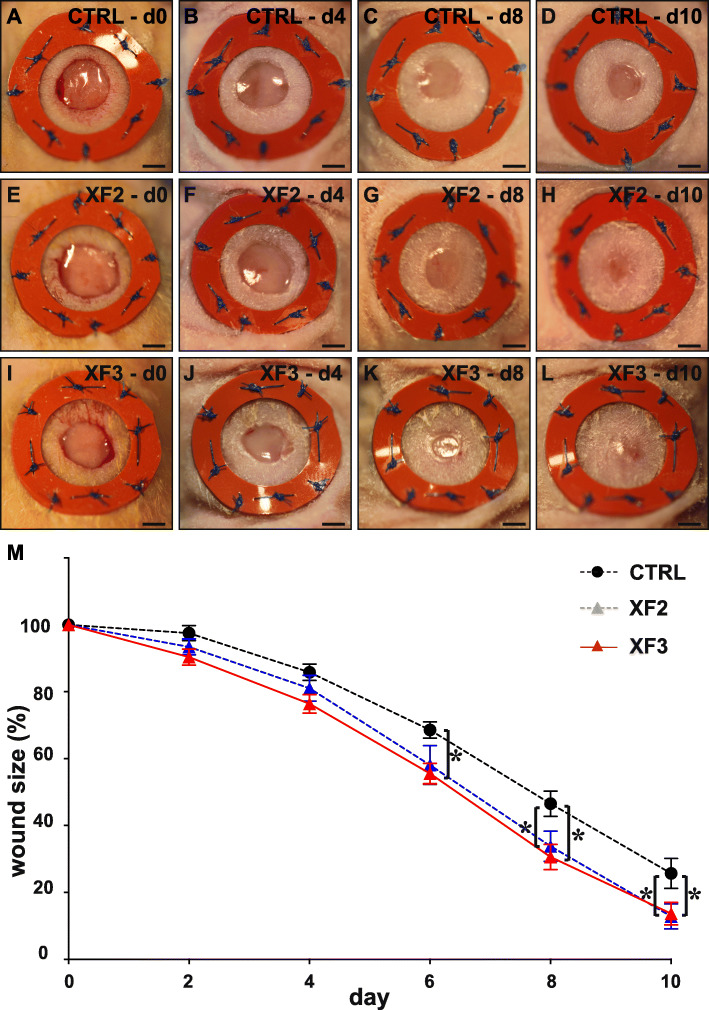
XF-hMAPC cells modestly accelerated wound closure by contraction. Brightfield overview images of the splinted wound on day (d) 0 (**a**, **e**, **i**), d4 (**b**, **f**, **j**), d8 (**c**, **g**, **k**), and d10 (**d**, **h**, **l**) after wounding of mice treated with Plasma-Lyte control (“CTRL”; **a**–**d**; black circles in **m**; *n* = 15), middle-dose XF-hMAPC cells (“XF2”; **e**–**h**; blue triangles in **m**; *n* = 16), or high-dose XF-hMAPC cells (“XF3”; **i**–**l**; red triangles in **m**; *n* = 16) and corresponding quantification in **m**. Data represent mean wound size expressed as % versus size at d0 ± S.E.M. (*P < 0.05 versus indicated condition by repeated-measures ANOVA). Scale bars: 2 mm in **a**–**l**

The wound healing process not only involves epidermal repair but also implies restoration of the dermal matrix layer, which is part of the late healing response [20]. Therefore, a second set of mice was followed-up longer-term until 10 days after wounding, thereby only using the two highest XF doses (as the lowest XF1 dose was ineffective in the early wound stages) and a Plasma-Lyte CTRL reference. Initial wound sizes measured immediately after wound infliction were similar across the different treatment conditions (expressed as % versus the inner area of the ring sutured around the wound: 33 ± 1 for CTRL (*n* = 15), 32 ± 1 for XF2 (*n* = 16), 33 ± 2 for XF3 (*n* = 16; *P* > 0.05 by one-way ANOVA). Also, beyond 5 days after wounding, compared to Plasma-Lyte CTRL, high doses of XF-hMAPC cells had a continuous—but rather modest—effect on accelerating wound closure by contraction (Fig. 5).

**Fig. 6:**
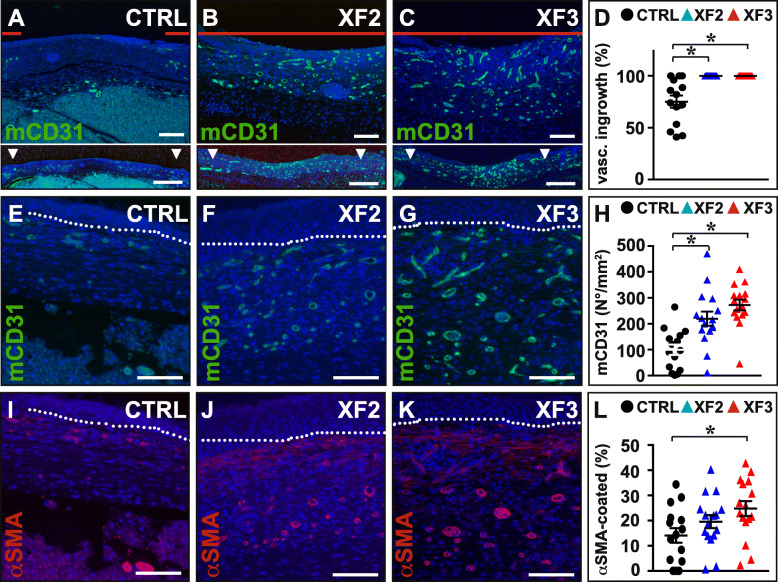
XF-hMAPC cells dose-dependently induced an elaborate and mature vascular network in the wound center late during wound healing. **a**–**d** Cross-sections representing the wound center stained for mouse (m)CD31 in green for the Plasma-Lyte control (“CTRL”; **a**; black circles in **d**; *n* = 14), middle-dose XF-hMAPC cell (“XF2”; **b**; blue triangles in **d**; *n* = 13), and high-dose XF-hMAPC cell (“XF3”; **c**; red triangles in **d**; *n* = 15) groups and the corresponding quantification in **d**. Data represent the vascular ingrowth relative to the wound length expressed as % ± S.E.M. (*P < 0.05 versus indicated condition by one-way ANOVA). Red horizontal lines in **a**–**c** indicate distance covered by the vascular fronts, white arrowheads in the overview panels in the bottom indicate wound borders. **e**–**h** Cross-sections zooming in on the wound center stained for mCD31 in green for CTRL (**e**; black circles in **h**; *n* = 15), XF2 (**f**; blue triangles in **h**; *n* = 16), and XF3 (**g**; red triangles in **h**; *n* = 16) groups and the corresponding quantification in **h**. Data represent the mean fraction of mCD31^+^ vessels expressed as number per area (in mm^2^) ± S.E.M. (*P < 0.05 versus indicated condition by one-way ANOVA). **i**–**l** Cross-sections (same as in **e**–**g**) zooming in on the wound center stained for α-smooth muscle-actin (αSMA) in red for CTRL (**i**; black circles in **l**; *n* = 15), XF2 (**j**; blue triangles in **l**; *n* = 16), and XF3 (**k**; red triangles in **l**; *n* = 16) groups and the corresponding quantification in **l**. Data represent the mean fraction of αSMA^+^ vessels expressed as % of mCD31^+^ vessels ± S.E.M. (*P < 0.05 versus indicated condition by one-way ANOVA). Dermo-epidermal junction is indicated by white dotted lines in **e**–**g** and **i**–**k**. DAPI was used as nuclear counterstaining (in blue) in **a**–**c**, **e**–**g**, and **i**–**k**. Magnifications at which pictures were taken: × 2.5 in **a**–**c** (bottom); × 10 in **a**–**c** (top); × 20 in **e**–**g**, **i**–**k**. Scale bars: 1 mm in **a**–**c** (lower panels), 500 μm in **a**–**c** (upper panels), 100 μm in **e**–**g** and **i**–**k**

At day 10 post-wounding, all XF-hMAPC-treated mice featured complete vascular ingrowth into the wound center, while in most CTRL-treated wounds there was still an avascular gap in the wound center (Fig. 6a–d). Accordingly, XF-hMAPC cells had a significantly increased CD31^+^ blood vessel density in the wound center (Fig. 6e–h), and like in the early stages, a significant fraction of these ingrowing vessels was stabilized by αSMA coating (Fig. 6i–l), both in a dose-dependent manner. Thus, XF-hMAPC transplantation significantly promoted the ingrowth and maturation of blood vessels in the wound center at later stages of the healing process.

**Fig. 7:**
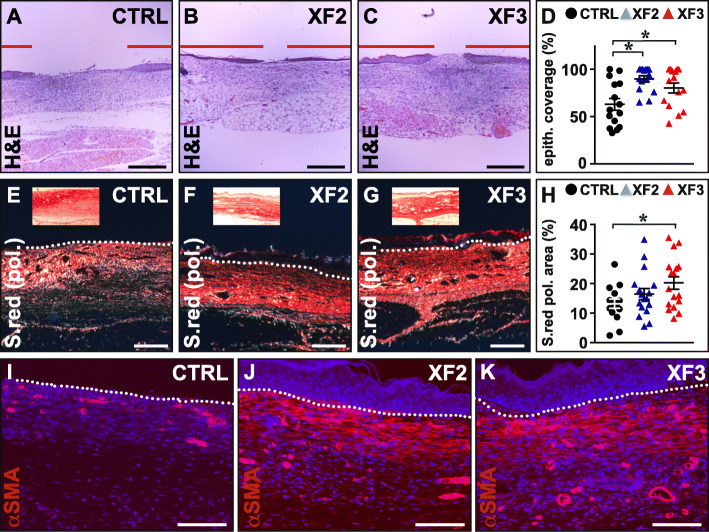
XF-hMAPC cells dose-dependently induced re-epithelialization and dermal collagen organization late during wound healing. **a**–**d**. Cross-sections zooming in on the center of the wound bed stained for hematoxylin/eosin (H&E) for the Plasma-Lyte control (“CTRL”; **a**; black circles in **d**; *n* = 15), middle-dose XF-hMAPC cell (“XF2”; **b**; blue triangles in **d**; *n* = 15), and high-dose XF-hMAPC cell (“XF3”; **c**; red triangles in **d**; *n* = 15) groups and the corresponding quantification in **d**. Data represent mean re-epithelialization distance from the wound edges relative to the wound length expressed as % ± S.E.M. (*P < 0.05 versus indicated condition by one-way ANOVA). Red horizontal lines in **a**–**c** indicate distance covered by the neo-epidermis. **e**–**h** Cross-sections stained for Sirius red (S.red) and photographed in polarized light (pol.) for the CTRL (**e**; black circles in **h**; *n* = 15), XF2 (**f**; blue triangles in **h**; *n* = 17), and XF3 (**g**; red triangles in **h**; *n* = 17) groups and the corresponding quantification in **h**. Data represent mean fractional red birefringent Sirius red^+^ area expressed as % ± S.E.M. (*P < 0.05 versus indicated condition by one-way ANOVA). Insets represent corresponding brightfield pictures. **i**–**k** Cross-sections stained for α-smooth muscle-actin (αSMA) in red for the CTRL (**i**), XF2 (**j**), and XF3 (**k**) groups showing significant myofibroblast accumulation in the wound center of cell-treated but not vehicle-treated wounds. Dermo-epidermal junctions are indicated by dotted white lines in **e**–**g** and **i**–**k**. Hematoxylin was used as nuclear counterstaining (in blue) in **a**–**c** and DAPI (in blue) in **i**–**k**. Magnifications at which pictures were taken: × 10 in **a**–**c**, **e**–**g**; × 20 in **i**–**k**. Scale bars: 400 μm in **a**–**c**, 200 μm in **e**–**g**, and 100 μm in **i**–**k**

XF-hMAPC transplantation also had a substantial effect on epithelial regeneration in the later stages of wound healing, as evidenced by nearly full epithelial coverage in the majority of mice in both XF-hMAPC dose groups in contrast to the mostly partial epithelial regeneration response in CTRL-treated wounds (Fig. 7a–d). Furthermore, while cell treatment did not significantly (*P* > 0.05 by one-way ANOVA) affect the amount of fibrillar collagen deposited (area % of Sirius red under brightfield microscopy: 48 ± 3 for CTRL (*n* = 15), 53 ± 2 for XF2 (*n* = 17), and 54 ± 3 for XF3 (*n* = 17)), the organization of the latter was significantly improved as evident from polarized light microscopy revealing a dose-dependent increase in the amount of red-birefringent (i.e., highly organized and crosslinked) fibers in the wound center (Fig. 7e–h; ratio red birefringent/total collagen in %: 13 ± 1 for CTRL (*n* = 15), 16 ± 2 for XF2 (*n* = 17), and 19 ± 2 for XF3 (*n* = 17; *P* < 0.05 versus CTRL by one-way ANOVA)). Remarkably, spatial distribution of αSMA^+^ myofibroblasts was altered by cell therapy, i.e., a larger fraction of the cell-treated wounds featured significant amounts of these myofibroblasts in the wound center (25, 56, and 50% for CTRL, XF2, and XF3, respectively; Fig. 7i–k). On the other hand, myofibroblast presence was not different in the wound edges at day 10 post-wounding. Thus, XF-hMAPC cells improved late wound healing by accelerating epithelial closure and increasing dermal collagen organization.

## Discussion

**Fig. 8:**
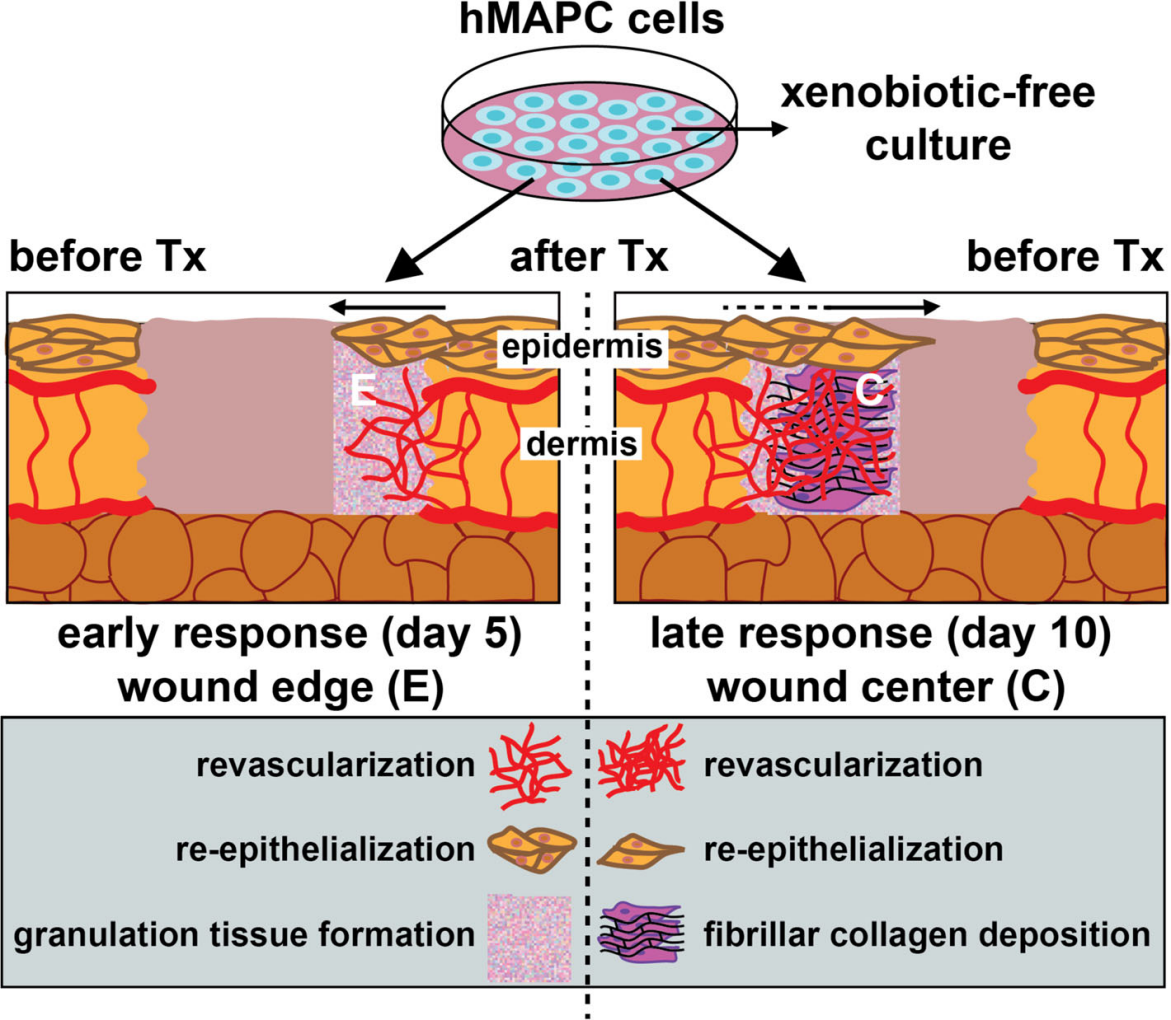
Graphical summary. Following their transplantation (Tx) in the wound bed, hMAPC cells cultured in xenobiotic-free media positively impact on the early (*left*) and late (*right*) healing process by improving both epidermal and dermal healing parameters in a multifactorial fashion

In this study, we investigated the efficacy of serum-free-, XF-expanded hMAPC cells in a mouse wound healing model. In agreement with previous results using their non-GMP serum-cultured equivalents [10], we demonstrated that XF-hMAPC cells had a dose-dependent positive effect on macroscopic and microscopic healing parameters in a splinted full-thickness wound model, suggesting a possible therapeutic effect of these cells in various wound healing applications (Fig. 8).

We first demonstrated that XF-hMAPC cells induced blood vessel formation in an in vivo Matrigel plug assay. In order to examine whether this effect could be of therapeutic relevance, XF-hMAPC cells were tested for their ability to support neovascularization in a splinted wound model. In this model, positive effects of the cells were seen both on early and late stages of healing. While improving blood vessel growth both in early and late stages was an important asset of XF-hMAPC administration, this was likely due to their paracrine effect on the host vasculature, as the intrinsic in vivo capacity of these multipotent cells for endothelial differentiation is limited (this and other studies [10, 13, 21]). Micro-array studies on hMAPC cells grown in regular growth media have shown a superior pro-angiogenic expression profile compared to MSCs [13], yet the factors responsible for the superior pro-angiogenic effect remain to be determined. In a recent study by Ahangar et al., it was demonstrated that hMAPC-secreted factors had positive effects on skin cell proliferation, migration, collagen deposition, and the hMAPC secretome improved wound healing without the presence of MAPC cells [22]. This further supports the notion that the benefit of hMAPC cells is mostly related to their trophic effect on the healing process.

MSCs have been shown to cause excessive wound contraction by differentiating into myofibroblasts [23]. Wound closure by XF-hMAPC cells was mainly due to re-epithelialization, rather than closure by contraction, the latter which—if excessive—may cause problems with mobility when wounds are located around joints [24]. While the presence of αSMA^+^ myofibroblasts accumulating in the wound edges correlated with the degree of wound contraction in a previous study [17], here, we found that XF-hMAPC cells caused an increased number of these cells to accumulate in the wound center, which apparently did not lead to a dramatic increase in wound contraction. We therefore hypothesize that these centrally located cells rather than having a role in wound contraction may be responsible for matrix deposition and/or matrix organization, the latter which was more extensively observed in XF-hMAPC-treated wounds. In support of this hypothesis, others have shown that the absence of αSMA^+^ myofibroblasts in αSMA-deficient mice can lead to less collagen deposition and organization, while their presence was not an absolute requirement for wound contraction [25]. It remains to be determined whether the beneficial effect of XF-hMAPC cells on (epi) dermal healing is mostly indirect through their pro-angiogenic actions or whether they also mediate healing through direct paracrine communication with (myo) fibroblasts and keratinocytes, the main effector cells of dermal and epidermal healing, respectively.

Over the last decade, a large number of studies have reported on the beneficial use of stem cells for cutaneous wound repair in pre-clinical and—importantly—also in clinical studies as for instance for the treatment of diabetic foot ulcers [26, 27]. While multiple study designs have been considered in terms of types of wound model, cell type/source (e.g., autologous or allogeneic) or route of administration (e.g., topical or systemic), collectively, these studies demonstrate that stem cells have a positive effect on wound healing (reviewed in Kirby et al. and Isakson et al. [28, 29]). The positive effects on wound size and clinical parameters seen in these different studies correlated with increased capillary density, re-epithelialization, collagen thickness, and granulation tissue formation [28]. Here, we report similar beneficial effects of XF-hMAPC cells on all these wound healing parameters in a splinted wound model. Given this multi-parametric effect, it may be assumed that XF-hMAPC cells can have beneficial effects in a clinical setting as well (Fig. 8).

A broad variety of biomaterial-based technologies are currently in development for the improvement of ulcer healing or for skin reconstruction in many other contexts. Hyaluronic acid (HA) or collagen representing important extracellular matrix (ECM) components of the skin are often part of engineered wound dressings or skin substitutes (reviewed by Murray et al. [30]). ECM-based dermal substitutes have shown relevance in the treatment of various types of wounds such as chronic ulcers [31], post-traumatic skin injuries [32], but also in regenerative plastic surgery methods (reviewed by Gentile et al. [33]) as for instance in scalp repair after tumor resection [34, 35]. To stimulate the regenerative capacity of wound dressings, growth factors and cytokines are incorporated, frequently in the form of autologous platelet-rich plasma (PRP), which also has been shown clinical potential for wound healing when used alone [36, 37]. Promising results in ulcer treatment have already been obtained in clinical studies combining PRP with HA [38–40]. Importantly, the above-mentioned biomaterials have been shown to also improve the performance of multiple stem cell types in skin repair, including bone marrow-derived MSCs and epidermal stem cells [41, 42]. Perhaps the combination currently most widely explored in clinical practice is the use of adipose tissue-derived stem cells with PRP [43–48]. As the nature (e.g., acute or chronic), severity (e.g., due to the presence of underlying disease), and location of wounds (e.g., in a region that requires hair regrowth) can be very diverse, the development of a broad variety of specialized tissue-engineering enhanced cell-based treatments (e.g., the use of adipose tissue-derived follicle stem cells for hair regrowth [49]) is a promising step in wound management and skin regeneration. On the other hand, the fact that some tissue-engineering approaches are commonly successful in different scenario’s such as the use of PRP in hair regrowth and wound healing suggests that these processes are driven by common biomolecular pathways involving common growth factors like VEGF, bFGF, platelet-derived growth factor, and epidermal growth factor (as discussed by Gentile et al. [50]).

In the current study, we locally applied XF-hMAPC cells in the skin gap immediately after wounding. In future studies, we may consider applying the cells into the granulation tissue later after wounding, as this situation is more representative for the clinical setting. Furthermore, like for other stem cell types, their potency may be enhanced by combining them with biomaterials. The MAPC-based therapeutic product, clinically developed as MultiStem® therapy, is being used in an allogeneic off-the-shelf setting, as the cells can be expanded ex vivo to obtain very large amounts of cells. Many of the published studies on the use of MSCs in wound healing have used autologous cells. However, the proliferative capacity of cells may decrease with donor age, while it can also not be excluded that the therapeutic activity of autologous cells from older donors who often have many risk factors, such as diabetes or hyperlipidemia, may be compromised [51]. It may thus be therapeutically as well as economically advantageous to develop a scalable standardized allogeneic cell product with demonstrated clinical activity. Having such a cell product free of serum and other animal-derived substances represents an important step towards broad clinical implementation of cell therapy. Here, we have shown that hMAPC cells derived and grown under such conditions from a healthy donor, and expandable at high scale without losing potency [15], perform well in promoting wound healing.

Although multiple studies have described positive effects of stem cells on wound healing, few of them have made use of cells expanded in serum-free medium. Sabapathy et al. published their findings that Wharton’s Jelly-derived MSCs cultured in serum-free media increased regeneration of a skin injury in mice [52]. Serum-free expanded autologous epidermal cells were shown to establish closure of a chronic wound in a patient [53]. Thus, the availability of data using serum-free cells in (pre)-clinical wound healing studies is still scarce. Our study is therefore one of the first to demonstrate that a serum-free expanded allogeneic stem cell product with high capacity for scalability in GMP settings has beneficial effects in wound healing.

## Conclusion

In conclusion, the results presented in this study demonstrate that XF-hMAPC cells significantly and dose-dependently improved multiple wound healing parameters both early and late during the healing process, making them appealing candidates for wound treatment in the clinical setting.

## Data Availability

Data sharing is not applicable to this article as no datasets were generated or analyzed during the current study.

## Abbreviations

MAPC: Multipotent adult progenitor cells
XF: Xenobiotic-free
MSC: Mesenchymal stromal cell
Rh: Recombinant human
VEGF: Vascular endothelial growth factor
bFGF: Basic fibroblast growth factor
PBS: Phosphate-buffered saline
CD: Cluster of differentiation
SMC: Smooth muscle cell
αSMA: α-smooth muscle-actin
PCK: Pancytokeratin
H&E: Hematoxylin/eosin
GMP: Good manufacturing practice
CTRL: Control
ECM: Extracellular matrix
PRP: Platelet-rich plasma
HA: Hyaluronic acid

## References

[1] Eming SA, Martin P, Tomic-Canic M (2014). Wound repair and regeneration: mechanisms, signaling, and translation. Sci Transl Med.

[2] Gurtner GC, Werner S, Barrandon Y, Longaker MT (2008). Wound repair and regeneration. Nature..

[3] Shaw TJ, Martin P (2009). Wound repair at a glance. J Cell Sci.

[4] Hendrickx B, Den Hondt M, Verdonck K, Vranckx JJ, Luttun A, Danquah MK, Mahato RI (2013). Cell and gene transfer strategies for vascularization during wound healing. Emerging trends in cell and gene therapy.

[5] Kovacsovics-Bankowski M, Streeter PR, Mauch KA, Frey MR, Raber A, van't Hof W (2009). Clinical scale expanded adult pluripotent stem cells prevent graft-versus-host disease. Cell Immunol.

[6] DePaul MA, Palmer M, Lang BT, Cutrone R, Tran AP, Madalena KM (2015). Intravenous multipotent adult progenitor cell treatment decreases inflammation leading to functional recovery following spinal cord injury. Sci Rep.

[7] Walker PA, Shah SK, Jimenez F, Gerber MH, Xue H, Cutrone R (2010). Intravenous multipotent adult progenitor cell therapy for traumatic brain injury: preserving the blood brain barrier via an interaction with splenocytes. Exp Neurol.

[8] Eggenhofer E, Popp FC, Mendicino M, Silber P, Van’t Hof W, Renner P (2013). Heart grafts tolerized through third-party multipotent adult progenitor cells can be retransplanted to secondary hosts with no immunosuppression. Stem Cells Transl Med.

[9] Aranguren XL, Pelacho B, Penuelas I, Abizanda G, Uriz M, Ecay M (2011). MAPC transplantation confers a more durable benefit than AC133+ cell transplantation in severe hind limb ischemia. Cell Transplant.

[10] Beerens M, Aranguren XL, Hendrickx B, Dheedene W, Dresselaers T, Himmelreich U (2018). Multipotent adult progenitor cells support lymphatic regeneration at multiple anatomical levels during wound healing and lymphedema. Sci Rep.

[11] LoGuidice A, Houlihan A, Deans R (2016). Multipotent adult progenitor cells on an allograft scaffold facilitate the bone repair process. J Tissue Eng.

[12] Cunha JP, Leuckx G, Sterkendries P, Korf H, Bomfim-Ferreira G, Overbergh L (2017). Human multipotent adult progenitor cells enhance islet function and revascularisation when co-transplanted as a composite pellet in a mouse model of diabetes. Diabetologia..

[13] Roobrouck VD, Clavel C, Jacobs SA, Ulloa-Montoya F, Crippa S, Sohni A (2011). Differentiation potential of human postnatal mesenchymal stem cells, mesoangioblasts, and multipotent adult progenitor cells reflected in their transcriptome and partially influenced by the culture conditions. Stem Cells.

[14] Karnieli O, Friedner OM, Allickson JG, Zhang N, Jung S, Fiorentini D (2017). A consensus introduction to serum replacements and serum-free media for cellular therapies. Cytotherapy..

[15] Crabbe MA, Gijbels K, Visser A, Craeye D, Walbers S, Pinxteren J (2016). Using miRNA-mRNA interaction analysis to link biologically relevant miRNAs to stem cell identity testing for next-generation culturing development. Stem Cells Transl Med.

[16] Seaman WE, Sleisenger M, Eriksson E, Koo GC (1987). Depletion of natural killer cells in mice by monoclonal antibody to NK-1.1. Reduction in host defense against malignancy without loss of cellular or humoral immunity. J Immunol.

[17] Hendrickx B, Verdonck K, Van den Berge S, Dickens S, Eriksson E, Vranckx JJ (2010). Integration of blood outgrowth endothelial cells in dermal fibroblast sheets promotes full thickness wound healing. Stem Cells.

[18] Simons M, Alitalo K, Annex BH, Augustin HG, Beam C, Berk BC (2015). State-of-the-art methods for evaluation of angiogenesis and tissue vascularization: a scientific statement from the American Heart Association. Circ Res.

[19] Decker CG, Wang Y, Paluck SJ, Shen L, Loo JA, Levine AJ (2016). Fibroblast growth factor 2 dimer with superagonist in vitro activity improves granulation tissue formation during wound healing. Biomaterials..

[20] Okonkwo UA, DiPietro LA. Diabetes and wound angiogenesis. Int J Mol Sci. 2017;18(7):1419. 10.3390/ijms18071419. 10.3390/ijms18071419 PMC5535911 28671607

[21] Aranguren XL, McCue JD, Hendrickx B, Zhu XH, Du F, Chen E (2008). Multipotent adult progenitor cells sustain function of ischemic limbs in mice. J Clin Invest.

[22] Ahangar P, Mills SJ, Smith LE, Strudwick XL, Ting AE, Vaes B (2020). Human multipotent adult progenitor cell-conditioned medium improves wound healing through modulating inflammation and angiogenesis in mice. Stem Cell Res Ther.

[23] Badillo AT, Redden RA, Zhang L, Doolin EJ, Liechty KW (2007). Treatment of diabetic wounds with fetal murine mesenchymal stromal cells enhances wound closure. Cell Tissue Res.

[24] Kwan PO, Tredget EE (2017). Biological principles of scar and contracture. Hand Clin.

[25] Ibrahim MM, Chen L, Bond JE, Medina MA, Ren L, Kokosis G (2015). Myofibroblasts contribute to but are not necessary for wound contraction. Lab Investig.

[26] Guo J, Dardik A, Fang K, Huang R, Gu Y (2017). Meta-analysis on the treatment of diabetic foot ulcers with autologous stem cells. Stem Cell Res Ther.

[27] Maksimova N, Krasheninnikov M, Zhang Y, Ponomarev E, Pomytkin I, Melnichenko G (2017). Early passage autologous mesenchymal stromal cells accelerate diabetic wound re-epithelialization: a clinical case study. Cytotherapy..

[28] Isakson M, de Blacam C, Whelan D, McArdle A, Clover AJ (2015). Mesenchymal stem cells and cutaneous wound healing: current evidence and future potential. Stem Cells Int.

[29] Kirby GT, Mills SJ, Cowin AJ, Smith LE (2015). Stem cells for cutaneous wound healing. Biomed Res Int.

[30] Murray RZ, West ZE, Cowin AJ, Farrugia BL (2019). Development and use of biomaterials as wound healing therapies. Burns Trauma.

[31] De Angelis B, Orlandi F, Morais D'Autilio MFL, Di Segni C, Scioli MG, Orlandi A, et al. Vasculogenic chronic ulcer: tissue regeneration with an innovative dermal substitute. J Clin Med. 2019;8(4):525. 10.3390/jcm8040525. 10.3390/jcm8040525 PMC6518262 30999579

[32] De Angelis B, Orlandi F, Fernandes Lopes Morais D’Autilio M, Scioli MG, Orlandi A, Cervelli V, et al. Long-term follow-up comparison of two different bi-layer dermal substitutes in tissue regeneration: clinical outcomes and histological findings. Int Wound J 2018;15(5):695–706. 10.1111/iwj.12912 PMC7949690 29590523

[33] Gentile P, Scioli MG, Bielli A, Orlandi A, Cervelli V (2017). Concise review: the use of adipose-derived stromal vascular fraction cells and platelet rich plasma in regenerative plastic surgery. Stem Cells.

[34] Bernstein JL, Premaratne ID, Levy AS, Kuhel WI, Kutler DI, Spector JA (2020). Reconstruction of full thickness scalp defects in extremely elderly patients using dermal regeneration templates. J Craniofac Surg.

[35] De Angelis B, Gentile P, Tati E, Bottini DJ, Bocchini I, Orlandi F (2015). One-stage reconstruction of scalp after full-thickness oncologic defects using a dermal regeneration template (Integra). Biomed Res Int.

[36] Milek T, Nagraba L, Mitek T, Wozniak W, Mlosek K, Olszewski W (2019). Autologous platelet-rich plasma reduces healing time of chronic venous leg ulcers: a prospective observational study. Adv Exp Med Biol.

[37] Suthar M, Gupta S, Bukhari S, Ponemone V (2017). Treatment of chronic non-healing ulcers using autologous platelet rich plasma: a case series. J Biomed Sci.

[38] De Angelis B, D'Autilio M, Orlandi F, Pepe G, Garcovich S, Scioli MG, et al. Wound healing: in vitro and in vivo evaluation of a bio-functionalized scaffold based on hyaluronic acid and platelet-rich plasma in chronic ulcers. J Clin Med. 2019;8(9): 1486. 10.3390/jcm8091486. 10.3390/jcm8091486 PMC6780765 31540446

[39] Nicoli F, Balzani A, Lazzeri D, Gentile P, Chilgar RM, Di Pasquali C (2015). Severe hidradenitis suppurativa treatment using platelet-rich plasma gel and Hyalomatrix. Int Wound J.

[40] Ramos-Torrecillas J, Garcia-Martinez O, De Luna-Bertos E, Ocana-Peinado FM, Ruiz C (2015). Effectiveness of platelet-rich plasma and hyaluronic acid for the treatment and care of pressure ulcers. Biol Res Nurs.

[41] Yang HY, Fierro F, So M, Yoon DJ, Nguyen AV, Gallegos A, et al. Combination product of dermal matrix, human mesenchymal stem cells, and timolol promotes diabetic wound healing in mice. Stem Cells Transl Med. 2020. p. 1-12. 10.1002/sctm.19-0380. 10.1002/sctm.19-0380 PMC7581456 32720751

[42] Yang R, Yang S, Zhao J, Hu X, Chen X, Wang J (2020). Progress in studies of epidermal stem cells and their application in skin tissue engineering. Stem Cell Res Ther.

[43] Alvaro-Afonso FJ, Sanz-Corbalan I, Lazaro-Martinez JL, Kakagia D, Papanas N. Adipose-derived mesenchymal stem cells in the treatment of diabetic foot ulcers: a review of preclinical and clinical studies. Angiology. 2020;3319720939467. 10.1177/0003319720939467 32723090

[44] Cervelli V, Bocchini I, Di Pasquali C, De Angelis B, Cervelli G, Curcio CB (2013). P.R.L. platelet rich lipotransfert: our experience and current state of art in the combined use of fat and PRP. Biomed Res Int.

[45] Cervelli V, Gentile P, De Angelis B, Calabrese C, Di Stefani A, Scioli MG (2011). Application of enhanced stromal vascular fraction and fat grafting mixed with PRP in post-traumatic lower extremity ulcers. Stem Cell Res.

[46] Cervelli V, Gentile P, Grimaldi M (2009). Regenerative surgery: use of fat grafting combined with platelet-rich plasma for chronic lower-extremity ulcers. Aesthet Plast Surg.

[47] Conese M, Annacontini L, Carbone A, Beccia E, Cecchino LR, Parisi D (2020). The role of adipose-derived stem cells, dermal regenerative templates, and platelet-rich plasma in tissue engineering-based treatments of chronic skin wounds. Stem Cells Int.

[48] Smith OJ, Jell G, Mosahebi A (2019). The use of fat grafting and platelet-rich plasma for wound healing: a review of the current evidence. Int Wound J.

[49] Gentile P. Autologous cellular method using micrografts of human adipose tissue derived follicle stem cells in androgenic alopecia. Int J Mol Sci. 2019;20(14):3446. 10.3390/ijms20143446. 10.3390/ijms20143446 PMC6678214 31337037

[50] Gentile P, Calabrese C, De Angelis B, Dionisi L, Pizzicannella J, Kothari A, et al. Impact of the different preparation methods to obtain autologous non-activated platelet-rich plasma (A-PRP) and activated platelet-rich plasma (AA-PRP) in plastic surgery: wound healing and hair regrowth evaluation. Int J Mol Sci. 2020;21(2). 10.3390/ijms21020431 PMC7014364 31936605

[51] Efimenko AY, Kochegura TN, Akopyan ZA, Parfyonova YV (2015). Autologous stem cell therapy: how aging and chronic diseases affect stem and progenitor cells. Biores Open Access.

[52] Sabapathy V, Sundaram B, MS V, Mankuzhy P, Kumar S (2014). Human Wharton’s Jelly mesenchymal stem cells plasticity augments scar-free skin wound healing with hair growth. PLoS One.

[53] Langa P, Wardowska A, Zielinski J, Podolak-Popinigis J, Sass P, Sosnowski P (2018). Transcriptional profile of in vitro expanded human epidermal progenitor cells for the treatment of non-healing wounds. J Dermatol Sci.

